# Prevention and early intervention in youth mental health: is it time for a multidisciplinary and trans-diagnostic model for care?

**DOI:** 10.1186/s13033-020-00356-9

**Published:** 2020-03-24

**Authors:** Marco Colizzi, Antonio Lasalvia, Mirella Ruggeri

**Affiliations:** 1 Section of Psychiatry, Department of Neurosciences, Biomedicine and Movement Sciences, University of Verona, 37134 Verona, Italy; 2 Department of Psychosis Studies, Institute of Psychiatry, Psychology and Neuroscience, King’s College London, London, SE5 8AF UK

**Keywords:** Youth mental health, Promotion, Prevention, Early intervention, Multidisciplinary care, Trans-diagnostic model

## Abstract

**Background:**

Similar to other health care sectors, mental health has moved towards the secondary prevention, with the effort to detect and treat mental disorders as early as possible. However, converging evidence sheds new light on the potential of primary preventive and promotion strategies for mental health of young people. We aimed to reappraise such evidence.

**Methods:**

We reviewed the current state of knowledge on delivering promotion and preventive interventions addressing youth mental health.

**Results:**

Half of all mental disorders start by 14 years and are usually preceded by non-specific psychosocial disturbances potentially evolving in any major mental disorder and accounting for 45% of the global burden of disease across the 0–25 age span. While some action has been taken to promote the implementation of services dedicated to young people, mental health needs during this critical period are still largely unmet. This urges redesigning preventive strategies in a youth-focused multidisciplinary and trans-diagnostic framework which might early modify possible psychopathological trajectories.

**Conclusions:**

Evidence suggests that it would be unrealistic to consider promotion and prevention in mental health responsibility of mental health professionals alone. Integrated and multidisciplinary services are needed to increase the range of possible interventions and limit the risk of poor long-term outcome, with also potential benefits in terms of healthcare system costs. However, mental health professionals have the scientific, ethical, and moral responsibility to indicate the direction to all social, political, and other health care bodies involved in the process of meeting mental health needs during youth years.

## Background

Promotion, prevention and early intervention strategies may produce the greatest impact on people’s health and well-being [1]. Screening strategies and early detection interventions may allow for more effective healthcare pathways, by taking action long before health problems worsen or by preventing their onset [2]. They also allow for a more personalized care in terms of tailoring health interventions to the specific sociodemographic and health-related risk factors as well as activating interventions specific to illness stage [3]. In this regard, the application of clinical staging models has been suggested to improve health benefits, by addressing the needs of people presenting at different stages along the continuum between health and disease [4]. Despite challenging, reformulating health services in this perspective may increase prevention and early intervention effectiveness, disease control and overall care, positively impacting on the health and well-being outcomes of a broader population [5]. Not to be overlooked, it may potentially reduce disease burden and healthcare system costs [6].

## The need for implementing prevention and early intervention in youth mental health

Prevention and early intervention are recognized key elements for minimizing the impact of any potentially serious health condition. However, while representing a field of remarkable achievement, that of early intervention in youth health is a target not completely accomplished yet [7]. This is particularly true for youth mental health. In fact, mental healthcare has been traditionally oriented to provide health benefits to adult populations during crisis events and major emergencies [8]. In this framework, mental health presentations to emergency settings in pediatric populations are somewhat frequent events [9]. Deinstitutionalization policies have only partially addressed this issue, also in light of the large variability worldwide in the implementation of community mental health services [10], especially for children and young adults [11].

Theoretical considerations about the opportunity to intervene in this specific age window in terms of mental health follow a number of evidence-based considerations. *First*, mental health is a key component of the person’s ability to function well in their personal and social life as well as adopt strategies to cope with life events [12]. In this regard, early childhood years are highly important, in light of the greater sensitivity and vulnerability of early brain development, which may have long-lasting effects on academic, social, emotional, and behavioral achievements in adulthood [13]. *Second*, most mental disorders have their peak of incidence during the transition from childhood to young adulthood, with up to 1 in 5 people experiencing clinically relevant mental health problems before the age of 25, 50% of whom being already symptomatic by the age of 14 [14]. Among people younger than 25 years old, mental health problems, especially anxiety and mood disorders, are the main cause of disability-adjusted life-years (DALYs), accounting for 45% of the global burden of disease, with problematic substance use including alcohol and illicit drugs being the main risk factor for incident DALY (9%) [15]. *Third*, most mental health services, as traditionally developed, have proven to be ineffective to provide healthcare during this critical period [16], with a modest use of mental health services despite the high prevalence of mental health problems among young individuals [17]. Also, following symptom onset, people aged 0–25 experience the greatest delay to initial treatment [18]. This is mainly due to two reasons. On one hand, young individuals, especially male, socioeconomically disadvantaged, and of ethnic minority, are less likely to establish initial contact with mental health services and stigma represents a major barrier in this regards [19]. When they do, they show high rates of disengagement [20]. On the other, significant delays in receiving care are also attributable to the reduced ability of services to rapidly deliver specialist mental healthcare for youth in need after a first primary care consultation [21]. When treatments are finally offered, the majority are not evidence-based [16].

Based on evidence summarized above, there is a pressing need to develop, or improve where present, youth mental healthcare models which can implement prevention and early intervention strategies. While progress has been made for psychotic disorders, also due to the successful application of an at-risk mental state concept [22], this is still largely unexplored in the context of common mental disorders, such as depression, anxiety, substance abuse, and eating disorders [23]. In order to meet the need for early intervention into childhood and young adulthood mental health difficulties, it is imperative to parallel redesign prevention and early intervention services for young populations, by promoting multidisciplinary collaborations between different specialized professionals in an enhanced and integrated service of extended primary care [5].

The aim of this narrative review is threefold: (i) to update on the current debate on the at-risk mental state concept and the possibility of widening the clinical area of intervention beyond psychotic disorders; (ii) to review the role of psychosocial difficulties early in life as potentially stable risk factors for poor mental health, and the extent to which they have been targets for early intervention; and (iii) to report on the progress made so far in implementing collaborative and integrated services for youth mental health within the healthcare system.

## Methods

The current literature review is intended to bring together research evidence on early life risk factors detection, youth mental health service provision, and application of a clinical staging model by using a trans-diagnostic approach. In particular, the present work aims to emphasize the relationship between these early intervention components and offer new directions for clinical research into the full development of a youth-based model of mental healthcare focused on prevention.

### Search strategy

A literature search was performed using electronic databases (MEDLINE, Web of Science, and Scopus), using a combination of search terms describing risk factors, clinical staging, and multidisciplinary prevention and early interventions in youth mental health. Special attention was given to available research of the past 25 years as a major transition in the clinical characterization of the prodromal phase of major psychiatric disorders in youth has occurred during the past 2 to 3 decades [21]. In addition, some research evidence gathered outside this search was reported, if considered appropriate by all authors.

### Eligibility criteria

Studies were eligible for inclusion in this review if assessing preventing strategies in youth in a trans-diagnostic and multidisciplinary approach. Studies were excluded in they (i) did not assess the application of a clinical staging model for youth mental health in a trans-diagnostic framework; (ii) did not investigate youth mental health service provision in a multidisciplinary framework; (iii) primarily assessed risk factors and preventive strategies in older populations rather than youth.

## Towards a trans-diagnostic clinical staging model to intercept a wider at-risk youth population

Over the nineteenth century, the so-called “prodromal state” (i.e. the period preceding the onset of severe mental disorders), was seen as a phase characterized by low-intensity or low-severity symptoms not sufficient to justify a categorical diagnosis, but whose ineluctable progression to full-blown disorder was only a matter of time. Towards the end of the last century, the formulation of the “at-risk mental state” concept [22] has represented a milestone in the development of a preventive approach to mental disorders, by overcoming the stagnant idea of inevitably ominous prognosis. This has dramatically loosened the deterministic approach to more severe mental disorders, such as schizophrenia, in favor of a more cautious approach to the potential future evolution of the condition in a psychosis-spectrum context where milder forms of the disorder and recovery are still possible. After a period of struggle to translate this paradigmatic advance in more effective mental healthcare practices, mostly because of the restrictive application of notions of “risk” and “transition” on the basis of positive psychotic symptom manifestation alone [24], we are finally facing a new turning point. Research evidence has increasingly recognized that, in addition to transition to psychosis, longer-term psychotic disorder, or persistent sub-threshold psychotic symptoms, progression to persistent mood, anxiety, personality and/or substance use disorders is also a very common outcome [25, 26]. This adds to the independent evidence that during development risk factors may contribute to a range of psychopathologies, and early indicators of later risk are often dimensional [27]. For instance, childhood adversities seem to impact negatively on a number of disorders [28]. Thus, in order to better characterize pluripotent and trans-diagnostic developmental processes and bio-behavioral mechanisms that give rise to mental illness, cross-disciplinary approaches need to integrate, if not overcome, the traditional diagnostic approach.

**Fig. 1:**
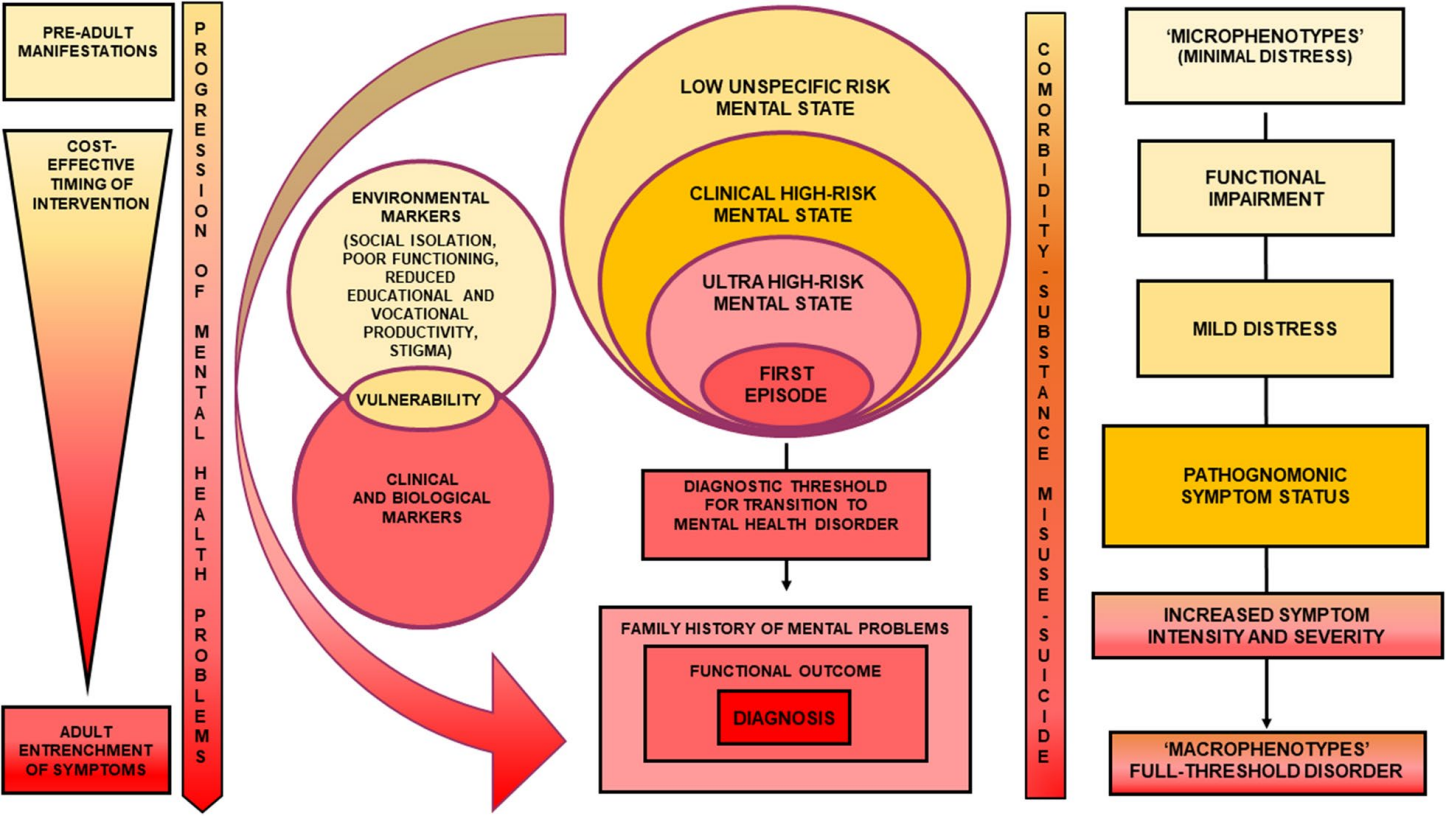
A trans-diagnostic clinical staging model to intercept a wider clinical high-risk mental state population

In this regards, integrated youth mental health services for people who are still in the earlier stages of a mental disorder may benefit from a wider clinical staging model framework far beyond the limited ultra-high risk (UHR) paradigm for psychosis. In particular, a trans-diagnostic clinical high-risk mental state (CHARMS) paradigm may increase capacity to intercept a wider range of lower risk cases than those with attenuated psychotic symptoms only, including people with sub-threshold bipolar and borderline personality symptoms as well as mild-moderate depression [22] (Fig. 1).

## Youth mental health: which targets for which interventions?

Neurodevelopmental changes occurring during youth make it a period of both vulnerability and opportunity for mental health. Research evidence indicates that a number of factors influence the person’s mental health from before birth until early adulthood, after which mental health can still be significantly modulated but to a lesser extent [29]. Meeting the child’s physical (i.e. healthy nutrition), psychological (i.e. stable and responsive attachment relationships), and social (i.e. supportive and safe environments) needs is key element to support optimal brain development, emotional regulation, and higher order cognitive function, with long-lasting health benefits [30]. Conversely, adversities during pregnancy and early childhood such as inadequate care, neglect, and trauma, have been shown to negatively impact on academic trajectories, psychosocial skills, physical resilience and the possibility of healthy aging [29, 31]. Also, depending on their nature, whether risk or protective factors, such environmental determinants may differentially modulate gene expression and stress response, with enduring health effects [32]. For instance, evidence from gene-environment interaction studies suggests that children carrying specific genetic variants are at increased risk for behavioral problems in later life, but only when raised in dysfunctional families [33]. Similarly, regardless their severity, stressful life events produce the most ‘toxic’ effect on children’s stress system, raising the risk of subsequent development of stress-related mental difficulties, when experienced in the absence of a stable and supporting environment [34]. In this context, it appears particularly relevant the development of a secure attachment between the child and a protective primary caregiver, in order to facilitate adaptive emotional and behavioral responses to stressful events [35]. In its absence, neurodevelopment may be undermined, making that person more vulnerable to further environmental insults and subsequent development of both internalizing [36] and externalizing [37] behavioral problems, including anxiety, depression, substance misuse, maladaptive eating patterns, sexual risk behavior, and suicidality. The relation between attachment difficulties and youth psychological problems is most likely bidirectional, such that problematic behaviors during childhood and adolescence may also precipitate difficulties in the caregiver-child/adolescent attachment bond, or exacerbate preexisting dysfunctional patterns [38]. Research has shown that internalizing and externalizing disorders of childhood are associated with an increased likelihood to develop a psychiatric disorder later in adulthood [39]. Interestingly, stringent tests of homotypic (a disorder predicting itself overtime) and heterotypic (different disorders predicting one another over time) prediction patterns suggest an increasingly developmentally and diagnostically nuanced picture, including but not limited to: (i) cross-prediction between anxiety and depression from adolescence to adulthood; (ii) adolescent oppositional defiant disorder, anxiety and substance disorders entirely accounting for the homotypic prediction pattern of depression overtime; and (iii) internalizing and externalizing psychopathology predicting psychosis-like experiences and vice versa [40]. Overall, these findings highlight how single disorder-oriented trajectories offer limited prospects for preventive interventions. Instead, interventions addressing multiple co-occurring problems are more likely to impact positively on youth mental health, potentially interrupting the continuity between childhood internalizing and externalizing psychopathology that may also co-occur with psychosis-like experiences on one hand, and psychiatric disorders in adulthood on the other. A large survey conducted by the World Health Organization (WHO) among 51,945 adults in 21 countries reported that eradication of childhood adversities, especially those associated with maladaptive family functioning (e.g. parental mental illness, child abuse, neglect), would lead to a 29.8% reduction of any mental disorder lifetime, and an even higher reduction when considering exclusively adolescence- (32.3%) and childhood-onset (38.2%) cases [28]. The possibility of preventing nearly one in two childhood-onset mental disorders is of crucial importance when considering that the experience of a mental disorder “kindles” a cascade of events which make recurrence later in life more likely [41]. Thus, promoting selective preventive strategies supporting children’s physiologic reactivity, cognitive control, and self-regulation through parenting- and classroom-based interventions, may represent a massive preventive action and ensure the earliest possible access to intervention with a view of limiting the continuity of mental health problems from childhood through to adolescence and adulthood.

**Fig. 2:**
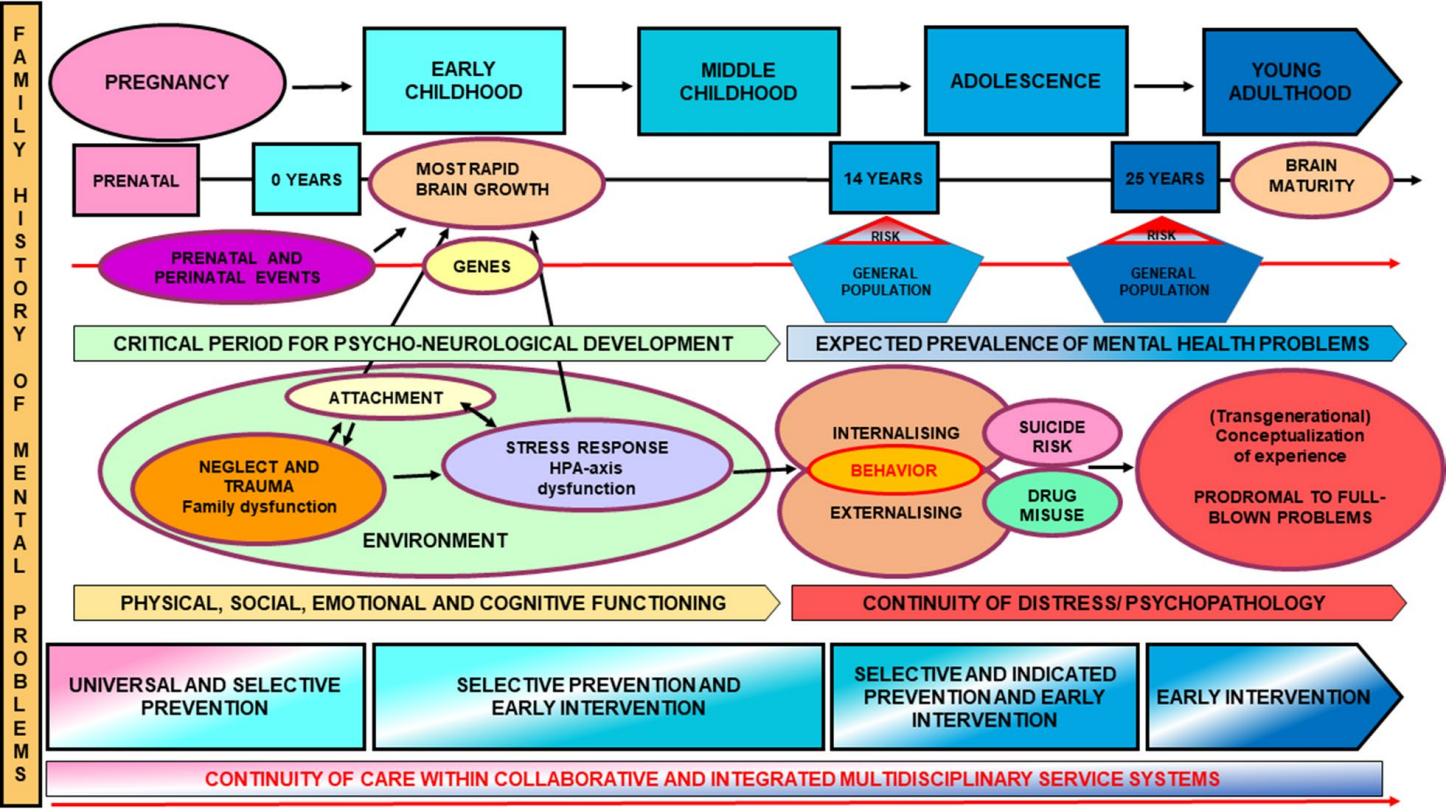
Summary of risk factors and pluripotent pathological trajectory for mental disorders

A summary of risk factors and pluripotent pathological trajectory for mental disorders encompassing the youth prevention and early intervention window is provided in Fig. 2.

## Mental health prevention and early intervention in youth: where is the evidence?

### Promotion of youth mental health

Mental health promotion focuses on enhancing the strengths, capacity and resources of individuals and communities to enable them to increase control over their mental health and its determinants. Prevention, on the other hand, aims to reduce the incidence, prevalence and severity of targeted mental health conditions [42]. In order to fill the treatment gap for mental, neurological, and substance use disorders worldwide, evidence-based guidelines developed by the WHO recommend that population level health interventions had an overall promotion focus. This is in line with the well-established continuum of care between interventions promoting positive mental health, interventions striving to prevent the onset of mental health disorders (primary prevention), and interventions aiming at early identification, case detection, early treatment, and rehabilitation (secondary and tertiary prevention) [43].

Meta-analytic work strongly supports the effectiveness of youth prevention programs addressing child abuse [44], negative consequences of parents’ divorce on children [45], substance abuse [46], and school-related problematic behaviors [47] in reducing rates of psychosocial difficulties later in life [48]. In this regard, multimodal preventing programs combining preschool intervention and family support have been associated to the most enduring beneficial effects on a number of social outcomes, including significant better overall academic performances and lower delinquency and antisocial behavior rates [49]. However, it is worth mentioning that promotion practices suffer from different mental health policies and social and contextual determinants. For instance, some health and social domains such as education, housing, nutrition, and healthcare, have pervasive influence on low income settings, while lack of supportive environments and community networks may have more detrimental effects in urban areas with high population density or ethnic minorities [50, 51]. Most likely, promotion programs require tailoring to the specific socio-cultural setting. Depending on its critical issues and what interventions are needed most, the implementation of effective programs goes through reorienting health services. Also, dialogue between health research, health professionals, health service institution, and governments is of paramount importance, especially to deliver integrated and multidisciplinary actions for the benefit of the entire community [50].

### Primary prevention in youth mental health

#### Developmental model for primary prevention

Primary prevention strategies may be universal, selective, or indicated, depending on whether they target the general population, a sub-group of the population, or specific individuals, respectively [42]. Rather than being separate, they should be seen as an integrated set of preventive interventions that continue throughout the neurodevelopmental stages of life as well as the intensification of risk [52].

#### Universal prevention (pre-clinical stage)

Mental health universal prevention aims at promoting normal neurodevelopment. Even though there is no consensus on which might be the pathophysiological mechanisms to be addressed during early development, promising findings suggest that developmental anomalies and behavioral deficits observed during childhood may be, at least partially, modifiable [53]. A number of effective pharmacological and psychosocial interventions for universal prevention have been identified, including: (i) perinatal phosphatidylcholine [54] and *N*-acetylcysteine [55] administration to support infants’ brain development and anti-inflammatory neuroprotection; (ii) lifetime omega-3 fatty acid [56–58], vitamin [57–59], sulforaphane [60], and prebiotic [61] supplementation to support good mental health by reducing neuroinflammation, oxidative stress, and microbiota dysbiosis; (iii) school-based behavioral interventions to minimize risk of bullying and peer rejection [62, 63] as well as substance abuse [64, 65]; (iv) exercise training to support brain plasticity [66], structure [67] and connectivity [68] as well as cognitive functioning [69].

#### Selective prevention (clinical stage 0)

Selective interventions aim at preventing the manifestation of psychiatric symptoms, thus altering the developmental pathway to full-threshold disorders in the premorbid state. Recipients of these interventions are individuals whose risk of developing a mental disorder is significantly higher than the rest of the population, while still being asymptomatic [42]. A number of risk factors have been identified, including parental mental illness [70], paternal age [71], maternal and obstetric complications of pregnancy [72, 73], season of birth [74], ethnic minority [75], immigration status [76], urban environment [77], infections [78], childhood adversities [28], vitamin D deficiency and malnutrition [79], low premorbid intelligence quotient [80], traumatic brain injury [81], and heavy tobacco [82] and cannabis use [83, 84].

It is worth reporting that most risk factors are shared across multiple mental disorders, suggesting the poor validity of boundaries between diagnostic categories, at least at this stage [85]. Also, while some risk factors are easily correctible (e.g. vitamin D deficiency) or technically preventable (e.g. cannabis use, infections), other require restructuring the role of the youth mental health professional as well employing a cadre of paraprofessionals to work more intensively with a large population of at-risk young individuals (e.g. childhood adversities), and for still others it is difficult to envisage programs ethically or practically sustainable (season of birth, urban environment) [86]. A few studies evaluated the effectiveness of prenatal and early infancy preventive programs for infants and children who may be socially disadvantaged or potentially at risk [87, 88]. Results supported long-term positive effects of nursing home visits to expectant mothers and their families in difficult social circumstances [87] as well as school educational interventions and home teaching to support low-income families and their preschool children [88] in reducing child abuse, neglect, and criminal behavior as well as improving the use of welfare and family socioeconomic status [87, 88].

To date, timing school-based mental health assistance, assertiveness training, and stress and anxiety management have the greatest chance to prevent maladaptive behavior and symptomatic manifestations [89]. Finally, while there is no clear research evidence favoring selective interventions in specific targeted populations, a promising strategy has been suggested to be the identification of those young individuals exposed to these risk factors who also have a family history of severe mental illness, in light of the per se higher genetic component for risk of mental disorders [90].

#### Indicated prevention (clinical stage 1)

Indicated interventions aim at the identification of those individuals at clinical high risk for the development of a mental disorder who are functionally impaired and no longer asymptomatic [42]. Psychosis studies have identified in the first 2 years following the manifestation of functional impairment a period of particular risk for transition to full-blown disorder [91], with about a third only in remission [92]. More recently, a shift towards a broader focus no longer confined to the psychosis risk identification has been suggested, in line with the increasingly clear evidence that pathways to mental disorders are pluripotent and trans-diagnostic [22]. This follows also the evidence that a so narrowed approach guarantees a limited detection, approximately 5%, even for those patients who will eventually develop a first episode of psychosis [93]. In this respect, complimentary evidence comes from a large meta-analysis that evaluated the impact of indicated preventive actions among 4470 at-risk students presenting with a range of problems including depression, anxiety, anger, general psychological distress, cognitive vulnerability, and interpersonal problems [94]. Intervention strategies included cognitive-behavioral, relaxation, social skills training, general behavior, social support, mindfulness, meditation, psychoeducational, acceptance and commitment therapy, interpersonal psychotherapy, resilience training, and forgiveness programs. Results suggested that indicated interventions have positive effects not only in reducing the presenting problem but also in improving other areas of psychosocial adjustment [94].

Indicated interventions are still preventive and aim at altering the trajectory of mental disorders. Research evidence suggests that the development of services for indicated prevention has met the objectives of strengthening service engagement, reducing the duration of untreated illness, and liaising with secondary prevention interventions [42]. In particular, reducing the duration of untreated illness has been robustly shown to impact positively on the outcome of first-episode psychosis and schizophrenia in many ways [95]. Increasing evidence suggests a similar effect for other psychiatric disorders including major depressive disorder, bipolar disorder, panic disorder, generalized anxiety disorder, and obsessive–compulsive disorder [96]. Importantly, as some pre-diagnostic symptoms and neurobiological correlates are not specific for psychosis [97] and some undesired outcomes, such as decreased social functioning, quality of life, and occupational performance, are shared across mental disorders [98, 99], a hybrid strategy has been suggested in at-risk states involving symptom relief coupled to a reduction of transition [97]. In particular, control of symptoms and self-control of emotion and behavior as well as programs targeting poor social problem solving, low quality of social support, interpersonal conflict, loneliness, and other social difficulties in at-risk states may reduce the risk of progression to any mental health disorder, including bipolar disorder and depression [97].

### Secondary prevention in youth mental health (clinical stage 2)

If patients progress to the manifestation of full-blown psychiatric symptoms, it is paramount to actively work towards securing early and possibly complete recovery, by reaching a clinical and functional remission state. Secondary preventive strategies and early intervention services aim at mitigating the occurrence of negative prognostic factors such as long duration of untreated illness, poor treatment response, poor psychosocial well-being and functioning, comorbid substance use, and high burden on patients’ families, with the final goal of preventing relapse or incomplete recovery [90]. In order to improve the effectiveness of early intervention in mental health, a Cochrane systematic review has confirmed the need for greater collaboration between primary care sector and specialist mental healthcare services [100]. In this regard, ‘consultation-liaison’ and ‘collaborative care’ models seem to work better than the so-called ‘replacement model’, where primary care physicians make simple referrals to mental health services [100], for a number of youth-onset psychiatric disorders including depression [101–104], psychosis [105–117], bipolar disorder [118, 119], and panic disorder [120, 121], with promising evidence for generalized anxiety disorder, social phobia [122], and somatoform disorders [123].

These multicomponent intervention programs involve the delivery of pharmacological and psychosocial interventions, as well as psychoeducation and skills training. However, disappointing evidence from studies of the effect of collaborative care on depression indicate that the clinical improvement may not be maintained after discontinuing the multidisciplinary treatment [101]. Thus, one may speculate that discharging young people to primary care or generic mental health services, which are not designed to assist young populations in the early stages of a mental disorder, is likely to result in the erosion of the initial advantages of the collaborative care, thus not changing the trajectory and outcome of the condition. In the absence of studies assessing the longer-term efficacy of such interventions, especially in preventing poor outcome, treatment disengagement, and relapse, caution is being called [90].

### Tertiary prevention in youth mental health (clinical stage 3)

Tertiary prevention represents the last opportunity to mitigate the impact of mental health problems in youth. In fact, following the manifestation of a first episode of acute psychiatric symptoms, some patients may not reach full recovery, being still symptomatic or functionally impaired. Tertiary preventive strategies aim at addressing treatment resistance, poor psychosocial wellbeing and functioning, comorbid substance use, and high burden on patients’ families, with the final goal of preventing multiple relapses and disease progression [90]. While the biological evidence for an association between multiple relapse and further deterioration is conflicting [124], research suggests detrimental psychosocial and functional consequences of each relapse [125, 126]. The absence of validated interventions to prevent multiple relapses highlights the limited protective effect of psychopharmacological treatments in the long-term, urging the development of new strategies to avoid chronicity (clinical stage 4).

A summary of promotion and preventive interventions in youth mental health is provided in Table 1.

**Table 1:** Promotion and preventive strategies in youth mental health

	Identified key target areas	Areas for further improvement and future objectives
Promotion	Promotion-prevention continuum	Address entire community
	Nutrition and health care	Integrated and multidisciplinary actions
	Housing and homelessness	Healthcare-community collaborations
	Child abuse	
	Negative consequences of parents’ divorce	
	Family support	
	Education and school-related problematic behavior	
	Addictive substance use/dependence	
	Personal skill development/management of stressful life events	
Primary prevention	Life-span continuum (Early stage-intensification of risk continuum)	
Universal	Brain development and anti-inflammatory neuroprotection (Phosphatidylcholine and *N*-acetylcysteine supplementation)	Pathophysiological mechanisms during early development
	Neuroinflammation, oxidative stress, and microbiota dysbiosis (Omega-3 fatty acid, vitamin, sulforaphane, and prebiotic supplementation)	
	Bullying and peer rejection (School-based behavioral interventions)	
	Substance abuse	
	Brain plasticity, structure, connectivity, and cognitive functioning (Lifetime exercise training)	
Selective	Parental mental illness	Poor validity of boundaries between diagnostic categories
	Paternal age	Lack of evidence-based selective interventions
	Maternal and obstetric complications of pregnancy	Youth with family history of severe mental illness (genetic risk)
	Season of birth	
	Ethnic minority	
	Immigration status	
	Urban environment	
	Infections	
	Childhood adversities, socio-financial disadvantage, maladaptive behavior (Nursing home visits, school-based interventions, home teaching)	
	Vitamin D deficiency and malnutrition	
	Low premorbid intelligence quotient	
	Traumatic brain injury	
	Heavy tobacco and cannabis use	
Indicated	Psychosis-risk state	Limited psychosis detection rate
	Service engagement and liaison with secondary intervention services	Pluripotent and trans-diagnostic risk state
	Duration of untreated illness	Multi-component symptom intervention
	Control of symptoms and self-control of emotion and behavior (Cognitive behavioral, relaxation, mindfulness, and meditation strategies)	
	Poor social problem solving and low quality of social support (Social skill training)	
	Interpersonal conflict (Interpersonal psychotherapy, forgiveness programs)	
	Loneliness and social difficulties in general (Resilience training)	
Secondary prevention	Collaborative care	Primary care-specialist mental health care collaborations
	Recovery	
	Duration of untreated illness	
	Poor treatment response/treatment resistance	
	Poor psycho-social well-being and functioning	
	Comorbid substance use	
	Burden on families	
Tertiary prevention	Recovery	Disease progression
	Poor treatment response/treatment resistance	Interventions to prevent multiple relapses
	Poor psycho-social well-being and functioning	
	Comorbid substance use	
	Burden on families	

## Towards the development of integrated and multidisciplinary services for the young population

Over the last decade, reforming youth mental health services in the perspective of integration and collaboration between different healthcare professionals has gained increasing interest [127]. Parallel, early intervention models, initially designed to assist people with psychotic disorders, have expanded their area of intervention to mood, personality, eating, and substance use disorders [128]. Thus, it has become increasingly possible to offer multidisciplinary and integrated healthcare to young people below the age of 25 with a variety of mental health difficulties as well as support their families.

In the USA, the Massachusetts Child Psychiatry Access Project (MCPAP) promoted the creation of a statewide service favoring collaborations between primary care practices and specialized child and adolescent psychiatry services. MCPAP has a wide area of intervention including attention deficit hyperactivity disorder, depression, anxiety as well as initial psychopharmacological treatment [129]. Studies have shown that most primary care practices have enrolled in the program, increasing young individuals’ access to psychiatric services and overall satisfaction [130]. With the aim of productively integrating and enhancing collaborative care at all levels of prevention, the Massachusetts Mental Health Services Program for Youth (MHSPY) has also implemented home-based integrated clinical interventions to assist severely impaired youth with mental, social, and substance use problems as well as their families in the community. Studies have shown benefits of MHSPY interventions in terms of higher psychosocial functioning and family satisfaction as well as lower burden on services and risk to self and others [131].

In Australia, a 2006 government-funded initiative led to creation of ‘Headspace’, a multidisciplinary and integrated service offering early intervention for 12–25-year-old people with emerging mental health difficulties. Headspace has a wide area of intervention including mental health, physical health, vocational and educational support, and substance use [132]. In a decade, thanks to the creation of ‘communities of youth services’ (CYSs), Headspace has seen growing the number of its centers from 10 to more than 110, granting access to services to about 100,000 young people per year, including vulnerable, marginalized, and at-risk groups [8]. An independent evaluation of Headspace has shown positive effects of the service in terms of reducing suicide ideation, self-harm, and number of absent school or work days [133].

This healthcare model is transferred to other countries at an increasingly rapid rate. In Ireland, services called ‘Headstrong’ and ‘Jigsaw’ have developed, proving to be effective in facilitating access to community care to people aged 12–25 with emerging mental health difficulties [134]. In the United Kingdom, a youth-based mental health service called ‘Youth space’ has implemented integrated health benefits for people aged 0–25 years in the Birmingham catchment area [135]. Similar models have been developed or under construction in Denmark, Israel, California, Canada (the ACCESS, Adolescent/young adult Connections to Community-driven Early Strengths-based and Stigma-free services), British Columbia (‘The Foundry’ model), and the Netherlands (@ease) [8]. Interestingly, research is following suit, with programs moving from the early identification of states immediately preceding psychosis onset in late adolescence or early adulthood to the investigation of earlier phases of illness in vulnerable children and younger adolescents (e.g. London Child Health and Development Study) [136].

In summary, a mix of services is offered among these models of care, in order to target mental health and behavior, situational problems, physical or sexual health, alcohol or other drugs use, and vocational issues. Depending on the presenting concern, the proportion of each delivered service can vary as well as the main service provider (general practitioner, psychologist, allied mental health etc.) and funding source [137]. Moreover, elements indicating best practice have been identified, including being highly accessible (affordable, convenient, timely, non-stigmatizing, flexible, inclusive, and awareness raising), acceptable (youth-friendly, confidential, respectful, engaging, responsive, competent, and collaborative), appropriate (early intervention focused, comprehensive, developmentally-appropriate, suitable to early stages of illness, suitable to complexity of presentation, evidence-based, and quality assured), and sustainable (community-embedded, integrated within a national network, effectively managed, advocate for young people’s wellbeing). These elements represent a framework to be used to inform future development, performance indicators, and standards of care [138].

**Fig. 3:**
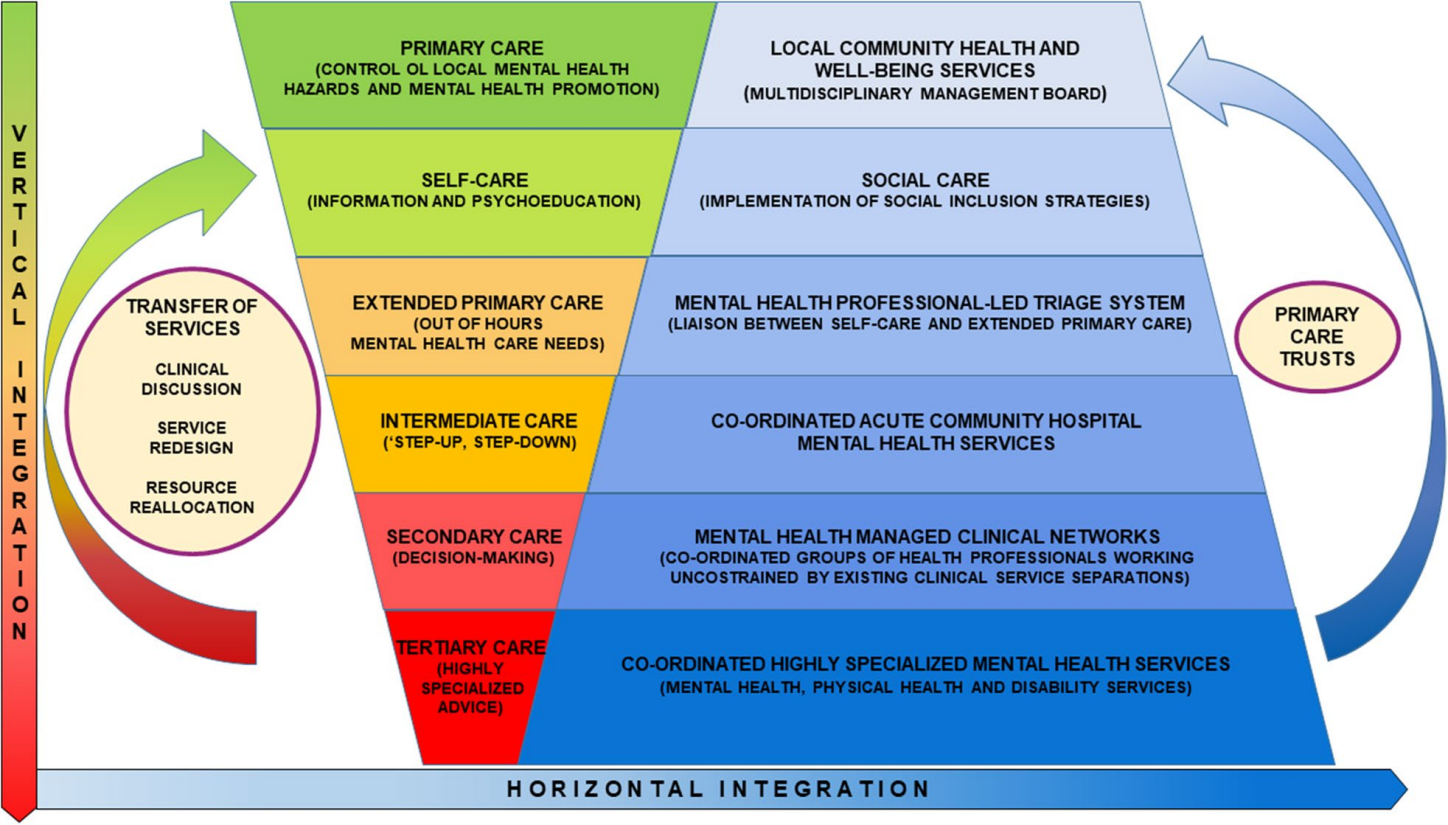
Vertical and horizontal integration of the enhanced model of primary care for mental health

Even though the topic is not covered in this reappraisal, for the sake of completeness Fig. 3 shows the next steps that would be required to vertically and horizontally integrate this enhanced model of primary care with more specialized and intensive services as well as other components of the health and social system.

## Conclusions and future directions

In order to guarantee youth a healthy mental development through promotion, prevention, and early interventions, research evidence supports the implementation of healthcare systems integrating mental, primary, and social care [128]. The recent implementation of mental health services for the 0–25 age span [8] poses new questions about what is needed now for this model of care to fulfill its potential. The continuity of youth mental health needs from an early age seems to go beyond the boundaries of what falls within the mental health professionals’ competences and duties, putting at stake the epistemological status of psychiatry. The mental health care sector has among its prerogatives the provision of effective interventions from early stages of illness to long-lasting conditions. However, it is increasingly clear how crucial is to deliver sustained early intervention across all potential stages, including the preclinical one, in order to avoid intermittent support and not to lose initial progresses. So, what do mental health professionals have to do? Medicalize potentially serious problems at the preclinical stage? Potentiate the social management of at-risk conditions? Both? In the mental health field, attempts of *reductio at unum* have left much to be desired in all ages, highlighting the greater complexity of the question. The recent debates about renaming mental health conditions or recognizing new ones on the basis of research evidence, far from being a mere hermeneutic or linguistic issue, underline the difficulty of managing what, through decades of clinical research, is emerging below the tip of the iceberg [139]. Promotion and prevention in mental health are not necessarily responsibility of mental health professionals alone. Research evidence summarized in this review suggests that health researchers and professionals as well as health service institutions and governments have to join forces to deliver integrated and multidisciplinary actions in mental health, especially in the early steps of the prevention chain. Mental health professionals have anyway the scientific, ethical, and moral responsibility to orient social, political, and overall health care actors involved in promotion and maintenance of mental health status.

## Data Availability

Not applicable.

## Abbreviations

ACCESS: Adolescent/young adult Connections to Community-driven Early Strengths-based and Stigma-free services
CHARMS: Clinical high-risk mental state
CYSs: Communities of youth services’
DALYs: Disability-adjusted life-years
MCPAP: Massachusetts Child Psychiatry Access Project
MHSPY: Massachusetts Mental Health Services Program for Youth
UHR: Ultra-high risk
USA: United States of America
WHO: World Health Organization
